# Mastering the difficulties presented by the peculiarities of island life. A commentary on: ‘Reconstruction of the spatio-temporal diversification and ecological niche evolution of *Helianthemum* (Cistaceae) in the Canary Islands using genotyping-by-sequence data’

**DOI:** 10.1093/aob/mcab025

**Published:** 2021-03-23

**Authors:** Mark Carine

**Affiliations:** Department of Life Sciences, The Natural History Museum, Cromwell Road, London, SW7 5BD, UK

**Keywords:** Helianthemum (Cistaceae), Canary Islands, diversification

## Abstract

This article comments on:

Rafael G. Albaladejo, Sara Martín-Hernanz, J. Alfredo Reyes-Betancort, Arnoldo Santos-Guerra, María Olangua-Corral and Abelardo Aparicio Reconstruction of the spatio-temporal diversification and ecological niche evolution of *Helianthemum* (Cistaceae) in the Canary Islands using genotyping-by-sequencing data, Annals of Botany, Volume 127, Issue 5, 16 April 2021, Pages 597–611, https://doi.org/10.1093/aob/mcaa090

Islands and island-like environments provide us with some of the most outstanding model systems for studying evolution. In 1902, Alfred Russell Wallace went so far as to suggest that ‘… it is not too much to say that when we have mastered the difficulties presented by the peculiarities of island life we shall find it comparatively easy to deal with the more complex and less clearly defined problems of continental distribution.’ (Wallace, 1902). Wallace’s view on the significance of islands for understanding biological processes remains a pervasive one today.

Oceanic archipelagos such as the Canary Islands, the subject of the paper in this issue by Albaladejo *et al*. (2021), have proved particularly attractive to evolutionary biologists and biogeographers and for very good reason: they are typically young, they have relatively simple geological systems that harbour distinctive floras with high levels of endemism, and they provide some of the most spectacular examples of *in situ* evolutionary radiations.

The Canary Island archipelago comprises seven main islands that are all volcanic in origin and that emerged from the ocean between 1.1 (El Hierro) and 23 (Fuerteventura) million years ago. With the exception of the two easternmost islands of Lanzarote and Fuerteventura that were joined to form a single landmass during the last glacial maximum, the islands have never been connected to each other or indeed to the continent. Within the islands, the elevational range and prevailing winds combine to generate markedly different habitats within a small area; on Tenerife, for example, it is possible to move from sub-desert coastal scrub, through temperate rainforest and into sub-alpine desert over just a short distance. More than 680 endemic plant taxa have evolved in the islands, the vast majority of which are restricted to a single island and restricted in the habitats that they occupy. It is evident that geographical isolation – both between islands and within islands – and adaptation to different habitats have contributed to the diversification of the archipelago’s flora.

Albaladejo *et al*.’s paper (2021) is one of a growing number of ‘new generation’ studies that are employing next generation sequencing approaches to investigate diversification within rapidly diversifying Canary Island radiations (Mort *et al.*, 2015; Puppo *et al.*, 2015). Albaladejo *et al.* (2021) focus on the Canary Island endemic clade of *Helianhemum* section *Helianthemum* (Fig. 1), a group of 15 species distributed across all islands except the youngest and westernmost island of El Hierro. Sampling an impressive 71% of the known extant populations of the group and employing genotyping by sequencing to generate a dataset of in excess of 4000 loci, the authors examine the tempo of diversification of the group and the role of geographical and ecological speciation.

**Fig. 1.**
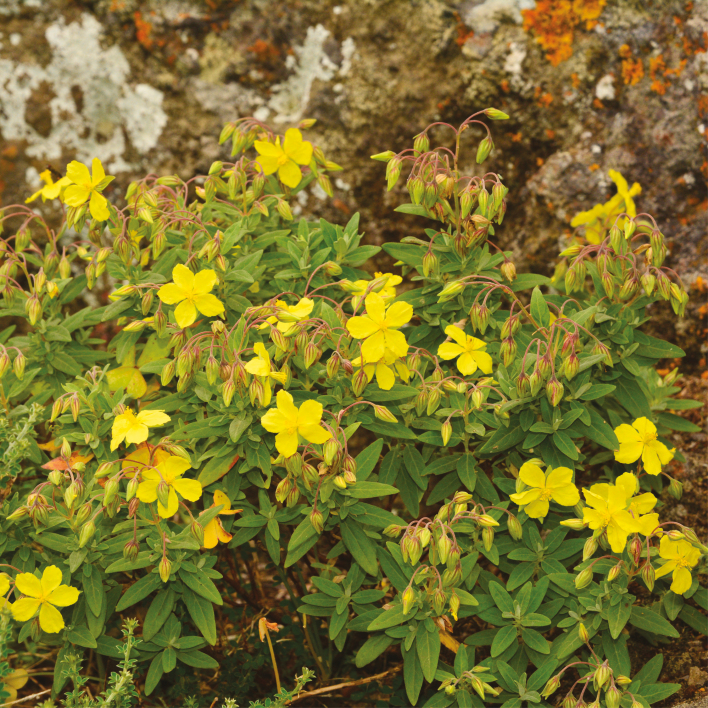
*Helianthemum broussonetii* Dunal in its native habitat on the Mesa del Brezal, Tenerife, Canary Islands, at 650 m. Image courtesy of Sara Martin-Hernanz.

It is now almost a quarter of a century since the first molecular phylogenetic papers on the Canary Island flora using Sanger sequencing were published (Bohle *et al.*, 1996; Francisco-Ortega *et al.*, 1996). They were followed by numerous similar studies that collectively examined a significant proportion of the archipelago’s endemic flora. We learned from those studies that there was typically strong support for the monophyly of the island lineage and for a Mediterranean sister group – a pattern consistent with a single colonization of the islands from the Mediterranean basin (Carine *et al.*, 2004). Within island clades, however, Sanger sequencing of a limited number of loci typically generated topologies in which most branches were extremely short and poorly supported. It was a pattern often interpreted as evidence for recent diversification but one that significantly limited inferences about within-archipelago diversification processes.

Albaladejo *et al.* (2021) demonstrate that *Helianthemum* conforms to the typical pattern of Canarian lineages in that it has a recent, probably Pleistocene, origin from the Mediterranean basin followed by extremely rapid diversification within the archipelago from the mid-Pleistocene onwards. Indeed, they show that the rate of speciation in *Helianthemum* seems to be particularly rapid when compared with other island groups. With a well-resolved topology for the island clade itself, they are also able to investigate how ecological shifts and island isolation contributed to the diversification of the group.

Their biogeographical analysis reveals Tenerife as the ancestral island for the Canary Island clade. Tenerife is the largest, tallest and ecologically most heterogeneous island in the archipelago but certainly not the oldest and nor is it the island closest to the continent; a simple stepping-stone model from near-continent to more remote islands is certainly not supported for *Helianthemum*. Analysis of the climatic niches reveals that the ancestral habitat for the group is mid-altitude with one shift upslope towards cooler and wetter habitats and another downslope towards warmer and drier conditions. The upslope shift occurred on Tenerife and was followed by westwards dispersal to a similar habitat on La Palma. The shift downslope to more xeric conditions occurred not on Tenerife but following dispersal to La Gomera, and the distribution and evolutionary history inferred for this group of species is particularly complex. There are four species in this group: three on the easternmost islands of Lanzarote (two species) and Fuerteventura (one species) and one on La Gomera. The results suggest that the group evolved following westwards dispersal from Gran Canaria to La Gomera, a dispersal event that bypassed Tenerife. Subsequent eastwards dispersal occurred from La Gomera to Fuerteventura and from there to Lanzarote resulting in the three eastern island endemics. This eastwards dispersal event bypassed both Tenerife and Gran Canaria. What explains such a pattern of ‘bypass dispersal’? The authors propose that the eastwards dispersal event may have been facilitated by the prevalence of westerly winds at times during the Pleistocene, but suitable climates almost certainly occur on the intermediate islands that were bypassed and that also demands explanation; extinction may also have contributed to the patterns observed. The extent to which the complex dispersal patterns observed in *Helianthemum* are common across Canarian plant groups will be only be revealed as similar integrative studies on other Canarian clades are undertaken.

It is notable that despite the large number of single nucleotide polymorphisms generated, not all nodes in the Canarian *Helianthemum* phylogeny are well supported. Indeed, for three clades, Albaladejo *et al.*. (2021) found evidence for a non-tree-like model of evolution. Hybridization is a common phenomenon in rapidly evolving Canary Island lineages within which reproductive barriers are often weak; indeed the islands offer one of the few well-characterized examples of homoploid hybrid speciation (White *et al.*, 2018). *Helianthemum* further supports the importance of combinatorial mechanisms (Marques *et al.*, 2019) – the reassembly of old genetic variants into novel combinations – as a process facilitating the rapid diversification of Canary Island lineages.

Wallace may have been correct in his view that ‘… the difficulties presented by island life are comparatively easy to deal with in comparison to continental areas’, but as this study by Albaladejo *et al.* (2021) reveals, the ways in which geographical isolation, ecological shifts and, indeed, hybridization have contributed to the diversification of island radiations are certainly not lacking in complexity.
